# Survey of healthcare-associated sink infrastructure, and sink trap antibiotic residues and biochemistry, in twenty-nine UK hospitals

**DOI:** 10.1016/j.jhin.2025.02.002

**Published:** 2025-02-15

**Authors:** G. Rodger, K.K. Chau, P. Aranega Bou, G. Moore, A. Roohi, The SinkBug Consortium, A.S. Walker, N. Stoesser

**Affiliations:** a Nuffield Department of Medicine, University of Oxford, Oxford, UK; b NIHR Health Protection Research Unit in Healthcare Associated Infections and Antimicrobial Resistance at University of Oxford in Partnership with Public Health England, Nuffield Department of Medicine, Oxford, UK; c Biosafety, Air and Water Microbiology Group, UK Health Security Agency, Porton Down, UK; d NIHR Oxford Biomedical Research Centre, Oxford University Hospitals NHS Foundation Trust, John Radcliffe Hospital, Oxford, UK

**Keywords:** Hospital-associated infection, Sinks, Sink drains, Antimicrobial resistance, Antibiotics, Sink design

## Abstract

**Background:**

Hospital sinks are linked to healthcare-associated infections. Antibiotics and chemicals in sink traps can select for pathogens and antimicrobial resistance (AMR). Optimizing sink design and usage can mitigate sink-to-patient dissemination of pathogens.

**Aim:**

To perform a large-scale survey of hospital sink infrastructure.

**Methods:**

Twenty-nine UK hospitals submitted photos and metadata for sinks across three wards (intensive care unit (ICU)/medical/surgical; January–March 2023). Photos were used to classify sink design as ‘optimal’ according to guidelines and published studies. Sink trap aspirates were dipstick-tested for antibiotics and chemistry. Logistic regression was used to characterize associations of ward type and sink location with optimal sink design or detectable trap antibiotics.

**Findings:**

Of 287 sinks surveyed, 111 were in ICUs, 92 in medical wards, and 84 in surgical wards; 77 were in medicines/drug preparation rooms, 97 on patient bays, 25 in patient side-rooms, and 88 in sluice rooms. Sink-to-bed ratios ranged from 0.23 to 2.83 sinks per patient bed and were higher on ICUs (1.21 versus 0.82 and 0.84 on medical and surgical wards, respectively; *P* = 0.04). The median sink-to-patient distance was 1.5 m (interquartile range: 1.00−2.21 m). Sink design varied widely; it was deemed ‘optimal’ for 65/122 (53%) sinks in patient bays/side-rooms and ‘optimal’ design was associated with side-room location (*P* = 0.03). Antibiotics were detected in 95/287 (33%) sink traps and were associated with medicines/drug preparation rooms (*P* <0.001). Sink trap chemicals detected included metals, chlorine, and fluoride.

**Conclusion:**

Sinks are common in hospitals, frequently close to patients, and often suboptimally designed. Commonly used antibiotics were detected in a third of sink traps and may contribute to the selection of pathogens and AMR in these reservoirs, and subsequent transmission to patients.

## Introduction

Sinks and sink drains have been associated with numerous healthcare-associated infection outbreaks, particularly of Enterobacterales, *Pseudomonas* spp., and other Gram-negative bacilli [1–3]. Accordingly, studies have estimated that between 7% and 40% of carbapenemase-producing Enterobacterales (CPE) acquisitions by hospitalized patients are attributable to a sink, likely due to splatter from contaminated drains [4,5]. Several studies implementing ‘water-less’ care have shown reductions in colonization and infection rates with these organisms [6–8]. However, removing sink infrastructure from hospitals may be challenging to implement and sustain. A promising alternative would be interventions which mitigate pathogen transmission from sinks by optimizing sink design and placement to limit the formation of pathogen biofilm and splatter from drains causing contamination of surrounding surfaces, items, staff, and patients [9].

Sink drains commonly act as a confluence of nutrients, chemicals such as soap and cleaning products, metals eluting from pipework, and patient waste (e.g. discarded body fluids), containing bacteria and drug metabolites, including antibiotic residues [10]. Sometimes, sinks are also used for direct disposal of antibiotics [11]. Water chemistry has also been shown to impact bacterial species composition [12,13]. The interplay between antibiotic exposures and water chemistry may therefore impose a complex selection pressure on bacteria residing in sink traps and associated pipework, favouring the exchange and spread of antimicrobial resistance (AMR) genes and emergence of multidrug-resistant organisms (MDROs) [5]. Therefore, it is essential to reduce pathogen and AMR gene selection by avoiding practices that contribute to the exposure of sink trap biofilms to nutrients, chemicals, and antibiotics [14]. A more informed and dynamic understanding of these selection pressures in sink traps, for example, by using rapid tests for chemicals and antibiotics in sink drains, could enable interventions designed to minimize behaviours that contribute to this kind of environmental contamination, such as inappropriate drug and/or waste disposal.

In the UK, several guidance documents set standards for sanitaryware including sinks in healthcare settings such as Health Technical Memoranda and Health Building Notes [15–19]. In addition, experimental studies have demonstrated that aspects of sink design and usage, such as tap placement over drains, drainage speed, and nutrient/antibiotic disposal, can all impact on selection and dissemination of MDROs from these reservoirs [14,20,21]. Basin fins have been shown to minimize splatter from taps hitting the basin surface, and may therefore represent a simple but beneficial design modification [22]. Opinions on the evidence supporting point of care filters on tap outlets are mixed, but some consider this a helpful approach to filtering incoming water [9].

Our primary research aim was therefore to characterize sink design features and determine whether they were consistent with current national best practice guidance and evidence across 29 UK hospitals. Our secondary aim was to use validated dipsticks to test sink drains for the presence of four antibiotic classes and evaluate chemical parameters that may represent important selection pressures for the emergence and transmission of drug-resistant pathogens [23].

## Methods

### Sink survey design and data collection

By advertising the research project through the National Infection Team Collaborative for Audit and Research (NITCAR), a national network of infection-associated healthcare professionals, we recruited 29 hospitals across the UK as part of a study of sinks and healthcare-associated infections (the Sink-Bug study; https://nitcollaborative.org.uk/wp/sinkbug/). At a single timepoint during a two-month window between January and March 2023, sites were asked to collect data on, and sink trap samples from, four sinks from a general critical care setting, three sinks from a general medical ward, and three sinks from a general surgical ward (10 sinks in total per hospital), surveying at least one patient bay sink and/or patient side-room sink, a sluice room sink, and a medicines/drug preparation room sink in each ward location. The total number of sinks and beds on a ward was counted, enabling the calculation of ward-level sink-to-bed ratios. For each sink, investigators collected data using an online survey designed in REDCap (https://www.project-redcap.org) and uploaded a photo of the sink enabling the study team to categorize sink design features centrally. Sink trap aspirates were collected using syringes and Ryles nasogastric tubes, and tested at the time of sampling with dipsticks on site which were also photographed [23]. Redcap data labels and data collected are represented in Supplementary datasets S1 and S2.

### Evaluation of sink infrastructures in patient bays and side-rooms

Specific sink features were classified from photos independently by two researchers and any disagreements resolved by discussion. The following were recorded: ward type, sink location within the ward (i.e. patient bay, patient side-room, sluice room, medicines/drug preparation room); sink basin material type, sink basin shape, presence of plug, overflow and drain strainer, location of drain with respect to tap outlet, presence of a basin fin; location of taps, method of tap operation, and shape of the outlet spout. For the sink design evaluation, the analysis included only sinks in patient bays or side-rooms where sink infrastructure is typically installed for the clinical purpose of handwashing, and where design aspects might pose the most immediate risk to patients.

The rationale for considering these specific sink design features was based on guidance and published evidence suggesting that minimizing biofilm formation, contamination, and splatter risk to patients and items would be beneficial (recommendations/evidence for specific features considered summarized in Supplementary Table S1). To achieve this, guidance suggests that sink basins should have an integral back outlet with connection to concealed services, no plug, no strainer, and no overflow. The drain-hole should not be positioned directly under the water outlet and should ideally be a horizontal drain-hole positioned at the back of the sink basin. Taps should be wall-mounted with levers or sensor taps, a single self-draining spout (fitted with a thermostatic mixing valve), and faucets that are not of the goose-neck variety as these do not empty after use [18]. For the purposes of this study, we therefore considered sinks with no plug, overflow or strainer; with a horizontal drain-hole position, wall-mounted taps with levers/sensors, and a single spout that was not a goose-neck spout, as being of ‘optimal’ design.

Given that items placed around the sink can be contaminated by water splatter (including bacteria) from the sink, site investigators were asked to document whether there was evidence of water splatter from the sink when the tap was turned on during a typical handwashing event. In addition, as contaminated splatter from sink drains has been shown to extend experimentally up to 1.5–2 m from the sink, and patients would therefore be at risk of contamination within this zone, investigators were also asked to record the distance of each surveyed sink to the nearest patient bed (in metres) [20,24].

### Antibiotic residue evaluations in sink trap aspirates

For the evaluation of antibiotic residues in sinks, we analysed all sinks surveyed, including sinks in sluice rooms and medicines/drug treatment rooms not evaluated as part of the infrastructure analysis above. The rationale was that the selection of antibiotic resistance in planktonic bacteria and biofilms could be relevant in any reservoir, either leaving the hospital in hospital wastewater, being transmitted along pipe infrastructure through biofilm growth, or being transmitted within wards by staff and equipment shared between medicines/drug treatment room or sluice room facilities and patient bays and side-rooms [25–28].

Sink trap aspirates were collected and dipstick tested following a sampling protocol defined in a set of training videos, using a standardized sampling kit provided to investigators. The QuaTest BTSQ 4-in-1 rapid test kit (Ringbio, UK) was used to detect β-lactam, tetracycline, sulfonamide, and quinolone antibiotic class residues in sink trap aspirates, having validated this approach previously, where we defined limits of detection of 3, 10, 20, and 8 μg/L for ampicillin, doxycycline, sulfamethoxazole, and ciprofloxacin, respectively [23]. The dipsticks were validated for these drugs as they would be the most commonly used antibiotics in these classes used in hospital settings in the UK. Sites were asked to upload photos of the dipstick tests performed, and interpretation of presence/absence of each antibiotic class was done by a single research team member.

### Chemical evaluations in sink trap aspirates

For the evaluation of sink trap chemistry, we also analysed all sinks surveyed, as for the antibiotic testing. Photos of the dipsticks were also uploaded to RedCap, and interpretations carried out by the same single researcher. The Bebapanda Upgrade 14-in-1 dipstick was used, previously validated against serial dilutions of analytes, and shown to detect reliably the presence of: copper (limit of detection (LoD): 10 mg/L), chlorine (1 mg/L), nitrate (25 mg/L), nitrite (20 mg/L), hardness (25 mg/L), alkalinity (40 mg/L), lead (50 mg/L), iron (100 mg/L), fluoride (25 mg/L) [23]. For those analytes where the dipstick LoD was higher than the lowest value present on the manufacturer’s dipstick readout chart, we considered results below the LoD as ‘indeterminate’ [23]. Similarly, if the colour change on the dipstick could not be clearly interpreted by the research team from the uploaded photos, these were also deemed ‘indeterminate’.

### Statistical analysis and data visualization

For descriptive statistics, the Kruskal–Wallis test was used to evaluate whether median sink-to-bed ratios differed by ward type, and the χ^2^-test to evaluate whether the proportions of sink traps positive for antibiotic residues differed by ward and/or sink location categories. For the sink infrastructure evaluation and presence of any antibiotic on trap dipstick testing, we used logistic regression to test whether sinks categorized as optimal or presence of antibiotics on dipstick (binary outcomes) were associated with ward type (intensive care unit (ICU), medical or surgical), and sink location (e.g. patient bay or side-room) (multivariate model). Data summary, statistical analysis, and data visualization was done using R (version 4.4.0, April 24^th^, 2024), various packages (e.g. ggplot2, dplyr) implemented in the tidyverse package, and the ggpubr and patchwork packages.

## Results

We surveyed a total of 287 sinks across 29 UK hospitals, with 27 sites submitting data on 10 sinks, one site on nine sinks and one site on eight sinks. Across all hospitals, 111/287 (39%) sinks were sampled on intensive/high-dependency care wards (ICUs), 92/287 (32%) sinks were sampled on medical wards, and 84/287 (29%) sinks were sampled on surgical wards. Within these wards 97/287 (34%) sinks were in patient bays, 25/287 sinks (9%) in patient side-rooms, 77/287 (27%) sinks in medicines/drug preparation rooms and 88/287 (31%) in sluice rooms.

Total ward sink-to-bed ratios (including surveyed sinks and other sinks on the ward) ranged from 0.23 to 2.83 sinks/patient bed (median: one sink/patient bed (interquartile range (IQR): 0.67−1.49)), with a higher median ratio on ICU versus medical or surgical wards (1.21 sinks/patient bed versus 0.82 and 0.84 sinks/patient bed respectively; Kruskal–Wallis, *P =* 0.04). The median number of sinks on a ward was 20 (IQR: 15−27, range: 4–63).

### Sink infrastructure in patient areas

For the sink infrastructure evaluation, the 122/287 (43%) sinks that were surveyed in patient bays or side-rooms were considered. It was found that 113/122 (93%) sinks were of ceramic design, including 52/113 (46%) that were a Contour 21-type sink, specifically designed for clinical settings (i.e. with no tap or chainstay hole, or overflow); 9/122 (7%) sinks were made of stainless steel. Only 1/122 (0.8%) sinks had a plug and 2/122 (2%) an overflow, but 41/122 (34%) of drains had strainers. Approximately two-thirds of basins had the recommended drain-hole position at the back of the basin (83/122 (68%)), but in 20/122 (16%) cases the drain was positioned such that the tap discharged directly over the drain. Basin fins were present in 14/122 (11%).

Most taps were wall-mounted (104/122 (85%)), operated with levers (113/122 (93%)) or sensors (7/122 (6%)), and had single spouts (119/122 (98%)). However, 26/122 (21%) of spouts were of the goose-neck variety (not recommended). 12/122 (10%) of tap outlets had point-of-use filters.

**Figure 1:**
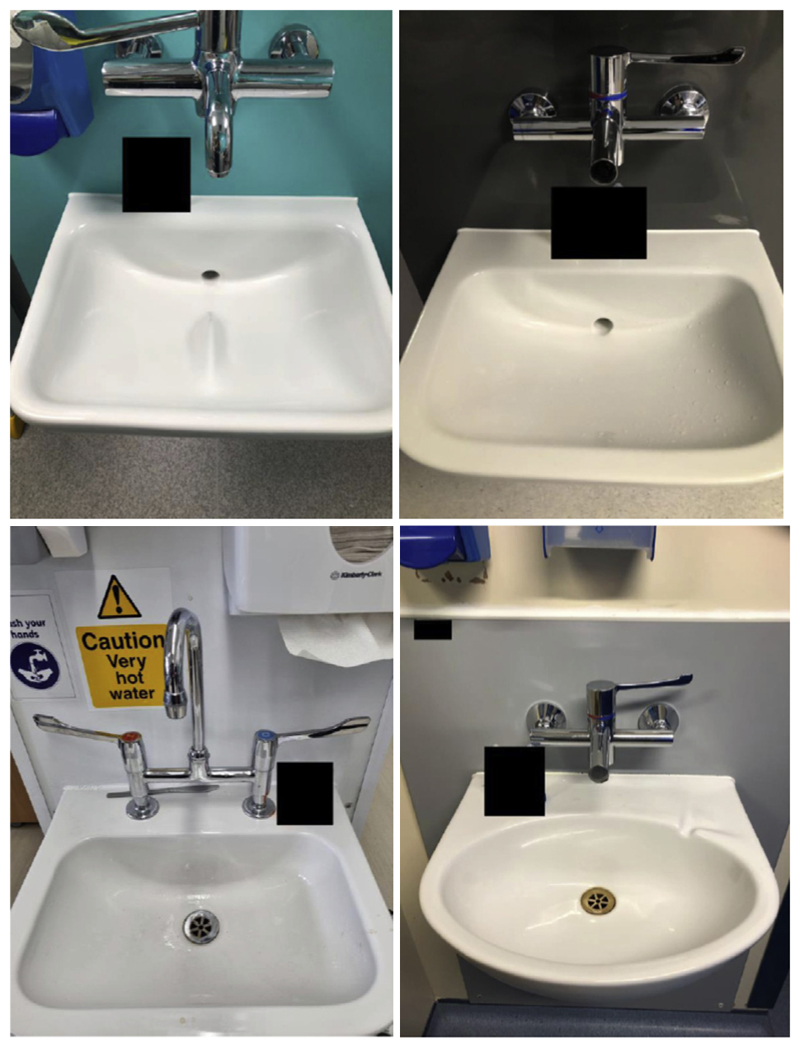
Examples of sink infrastructure deemed ‘optimal’ (upper panels) and ‘not optimal’ (lower panels). The black rectangles represent masks over labels that identified these sinks by hospital.

Based on designating clinical handwash sinks as ‘optimal’ if they had no plug or overflow or strainer, with a horizontal drain-hole position, wall-mounted taps with levers/sensors, and a single spout that was not a goose-neck spout (see Methods), 65/122 (53%) of sinks met these criteria and could be considered of ‘optimal’ design (Figure 1).

Considering ward type (medical, surgical, ICU) and sink location (patient bay or side-room), only patient side-room location was significantly associated with optimal sink infrastructure (as defined above) on multivariable analysis (odds ratio (OR): 2.96; 95% confidence interval (CI): 1.15−8.39; *P* = 0.03) (Table I).

With respect to splatter risk, for 15/122 (12%) sinks we observed patient or healthcare items were left immediately surrounding the basin, and in 41/122 (34%) of sinks splattering of the surroundings was observed when the taps were turned on by study staff simulating the approximate flow rate that would be used for a handwash. The median distance between the sink and the nearest patient bed edge was 1.5 m (IQR: 1.00−2.21).

### Sink trap antibiotic dipstick results

**Figure 2:**
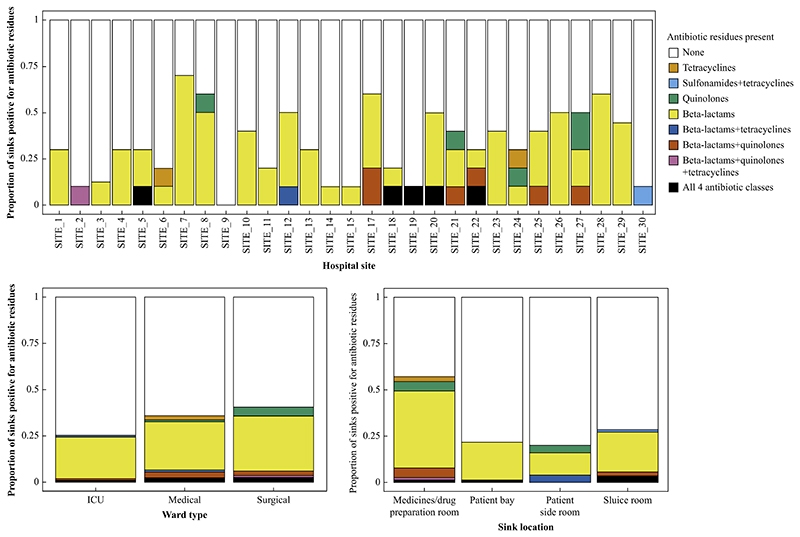
Proportion and location of sink traps positive for antibiotic residues, stratified by hospital site (upper panel; based on 10 sinks per site except eight sinks in site 3 and nine sinks in site 29); ward location (lower left panel; ICU (*N* = 111), medical wards (*N* = 92), surgical wards (*N* = 84)) and sink location (lower right panel; medicines/drug preparation room (*N* = 77), patient bay (*N* = 97), patient side-room (*N* = 25), patient sluice room (*N* = 88)).

Overall, amongst all sinks surveyed, 95/287 (33%) sink trap aspirates had at least one of four antibiotic classes detected, with 87/287 (30%), 17/287 (6%), 6/287 (2%), 10/287 (3%) of sink trap aspirates having detectable β-lactam, quinolone, sulfonamide, and tetracycline residues, respectively. One sink trap was positive for both sulfonamide and tetracycline residues (0.3%), one for β-lactam and tetracycline residues (0.3%), six for β-lactam and quinolone residues (2%), one for β-lactam and quinolone and tetracycline residues (0.3%), and five (2%) for all four antibiotic classes (Figure 2, upper panel).

Across ward types, 28/111 (25%) of sink traps were antibiotic-positive in ICU settings, versus 33/59 (56%) and 34/74 (46%) of sink traps in medical and surgical wards, respectively (χ^2^, *P* = 0.06) (Figure 2, lower left panel). Across sink locations, 44/77 (57%) of sink traps were positive in medicines/drug preparation rooms, 21/97 (22%) in patient bays, 5/25 (20%) in patient side-rooms, and 25/88 (28%) in sluice rooms (χ^2^, *P* <0.001) (Figure 2, bottom right panel).

On univariable analysis, ward type and sink location were both associated with the presence of antibiotic residues in sink traps (Table II). In a multivariable model including both ward type and sink locations, only sink location remained associated, with sink traps in sluice rooms, patient bays, and patient side-rooms less likely to have antibiotic residues than those in medicines/drug preparation rooms (adjusted ORs: 0.22 (95% CI: 0.11−0.42), 0.20 (95% CI: 0.06−0.57), and 0.30 (95% CI: 0.15−0.57) respectively; Table II).

### Sink trap biochemistry dipstick results

Sink trap water hardness, alkalinity and pH varied widely across sinks (Supplementary Figures S1, S2). Copper, lead, and silver were detected in some sink traps, as were fluoride and chlorine (Supplementary Figures S1, S2). No discernible patterns by ward type (Supplementary Figure S1) or sink location (Supplementary Figure S2) were observed.

## Discussion

This study systematically characterized sink infrastructure across common ward types (ITU, medical, and surgical wards) in 29 hospitals UK-wide, showing that sink design features vary substantially and, in comparison to national guidance and recent studies, are frequently inconsistent with what is currently considered the best design to minimize biofilm formation, water splatter, and dissemination of bacteria. In particular, in our survey, only 65/122 (53%) sinks in patient-associated bays/rooms demonstrated an ‘optimal’ design. Sinks in patient side-rooms were more likely to be of ‘optimal’ design (odds ratio: 2.96 (1.15−8.39; *P* = 0.03)), potentially because side-rooms are more likely to be associated with newer hospital builds or infrastructure refurbishment [29]. Although we focused on infrastructure evaluations in patient bays or side-rooms, similar risks from inappropriate sink infrastructure in terms of contaminating staff and equipment may also be relevant in other areas (e.g. common bathrooms, kitchenettes etc.).

Our study also demonstrated that antibiotic residues from antimicrobial classes routinely used in UK hospitals (i.e. β-lactams, fluoroquinolones, sulfonamides, and tetracyclines) were found in a third of sink p-traps surveyed, and may represent major selection pressures for the emergence of pathogens and AMR. These antibiotic residues are potentially related to the disposal of metabolized antibiotic in patient waste (e.g. discarded body fluids), or as unused drug. The latter is consistent with our finding that p-traps in medicines/drug treatment rooms were significantly (*P* <0.001) more likely to be positive for antibiotics than sink p-traps in other surveyed locations. Given the potential for biofilm to rapidly grow in connected pipework allowing migration of pathogens to sink drains within a ward, and the fact that equipment and staff may be contaminated by splatter during sink usage, this may represent a problem even if patients are not directly exposed to these sinks [27,30].

Biochemistry parameters such as pH/alkalinity and hardness, and the presence of metals and oxidizing agents such as chlorine and fluoride, varied markedly across the sinks surveyed, but without clear associations with ward type or sink location. A previous study with dense environmental sampling showed that genetic variation in carbapenemase-producing Enterobacterales in these reservoirs was associated with the specific niche, suggesting that the unique composition of selection pressures (such as antibiotics and chemicals) and microbiota in individual p-traps may be a key influence on which pathogens and AMR genes emerge and predominate in a given setting [5].

There were several limitations to this study. Only a small number of sinks across multiple wards and room locations per hospital (typically <15–20% of sinks) were sampled, not including sinks in certain ward settings (e.g. shared toilet facilities) or sinks outside of these settings (e.g. offices). A broader rather than denser sampling frame was chosen to support the generalizability of our findings, but this did restrict our power to test associations across numerous variables. Also, our survey was designed as a point prevalence study, without information on other relevant parameters such as the specifics of sink usage or local antibiotic use. Longitudinal evaluations of selection pressures in sink p-traps (e.g. such as how frequently the ‘predicted no effect concentrations’ (PNECs) for resistance selection are exceeded for antibiotics) would be of interest in relation to developing interventions, in addition to characterizing p-trap microbiomes and associations with infections and hospital-level AMR prevalence. The dipsticks used for testing are rapid, simple to use, and much cheaper than high-performance liquid chromatography, but only give a qualitative antimicrobial class-level readout rather than a drug-specific quantitative one. Identification of antibiotic classes not assayed by the dipstick or below its limits of detection may have been missed. Similarly, the biochemistry dipsticks generated a notable number of indeterminate readouts (30/122 (25%)), potentially because the chemicals being surveyed were present at levels below the limit of detection. It is important to note also that whilst we designated certain design features as ‘optimal’ based on guidance and supported by published studies, additional work would be of benefit on characterizing the impact of these features, other features such as sink trap type, and other novel designs in mitigating the risk sinks and contaminated splatter pose to patients, and we may not have captured all relevant features (Supplementary Table S1). In addition, some features, such as sink basin fins, which have been shown to mitigate splatter risk but are not part of current UK guidance, were not considered in our definition of ‘optimal’ in this study; making sink basin fins part of the ‘optimal’ definition would have resulted in <11% of sinks surveyed being designated as ‘optimal’.

To date, published data have shown consistently that sinks are a reservoir for some common healthcare-infection-associated pathogens, including MDROs, but optimal mitigation strategies remain unclear [1,2,31]. Recent systematic reviews have summarized the evidence relating to sink removal and other ‘water-free’ interventions in critical care settings, showing that although there are no randomized controlled trials and the quality and heterogeneity of these studies limits evaluation of interventions, ‘water-free’ care is associated with reductions in pathogen colonizations and healthcare-associated infections, particularly those associated with Gram-negative bacilli [32,33]. However, sink removal inhibits handwashing, which is known to be effective against other healthcare-associated pathogens such as *Clostridioides difficile* and norovirus, and can make personal care more difficult. Water-free care also relies on alternatives such as pre-packaged wipes, which not only have cost and environmental implications, but may themselves be implicated in outbreaks [34]. The most pragmatic approach may be to optimize sink usage, placement and design features to minimize the risk of bacteria and biofilm emerging into the basin and being disseminated by contaminated water splatter. In addition, limiting the presence of selection pressures such as antibiotics in sink p-traps through regular monitoring and optimizing sink usage and medication/waste disposal strategies may be beneficial. Our study shows that there is likely room for improvement across UK hospitals in both respects.

In conclusion, this study demonstrates that sink infrastructure across UK hospitals is highly variable, and that antibiotic residues are commonly found in sink p-traps. There is apparent scope for optimizing sink design to limit infection risk to patients from these reservoirs and the potential to reduce the exposure of bacterial communities in sink drains to antibiotics by altering disposal and sink usage practices – this could be monitored using a simple dipstick test.

## Supplementary Material

Supplementary data to this article can be found online at https://doi.org/10.1016/j.jhin.2025.02.002.

Database labels

Sink-associated metadata

Supplementary material

## Figures and Tables

**Table I:** Association between ward type and sink location with optimal sink infrastructure

Variable	No. of sinks	No. ‘optimal’ (%)	No. ‘not optimal’ (%)	Univariable OR (95% CI)	*P*-value	Multivariable OR (95% CI)	*P*-value
Ward type							
ITU	58	33 (57%)	25 (43%)	(reference)		(reference)	
Medical	33	15 (45%)	18 (55%)	0.63 (0.26–1.49)	0.30	0.57 (0.23–1.39)	0.22
Surgical	31	17 (55%)	14 (45%)	0.92 (0.38–2.23)	0.85	0.99 (0.41–2.45)	0.99
Sink location							
Patient bay	97	47 (48%)	50 (52%)	(reference)		(reference)	
Patient side-room	25	18 (72%)	7 (28%)	2.74 (1.09–7.58)	0.04	2.96 (1.15–8.39)	0.03

OR, odds ratio; ITU, intensive therapy unit.

**Table II:** Association between ward type and sink location with presence of antibiotics in sink traps

Variable	No. of sinks	Any antibiotic residue present	No antibiotic residue present	Univariable OR (95% CI)	*P*-value	Multivariable OR (95% CI)	*P*-value
Ward type							
ITU	111	28 (25%)	83 (75%)	(reference)		(reference)	
Medical	92	33 (36%)	59 (64%)	1.65 (0.91–3.05)	0.10	1.48 (0.78–2.82)	0.22
Surgical	84	34 (40%)	50 (60%)	2.02 (1.09–3.74)	0.02	1.72 (0.91–3.30)	0.10
Sink location							
Medicines/drug preparation room	77	44 (57%)	33 (43%)	(reference)		(reference)	
Patient bay	97	21 (22%)	76 (78%)	0.21 (0.11–0.40)	<0.001	0.22 (0.11–0.42)	<0.001
Patient side-room	25	5 (20%)	20 (80%)	0.19 (0.06–0.52)	<0.001	0.20 (0.06–0.57)	<0.001
Sluice room	88	25 (28%)	63 (72%)	0.30 (0.15–0.56)	<0.001	0.30 (0.15–0.57)	<0.001

OR, odds ratio; ITU, intensive therapy unit.
