# Supplementary material for: Survey of healthcare-associated sink infrastructure, and sink trap antibiotic residues and biochemistry, in twenty-nine UK hospitals
Source: J Hosp Infect. Author manuscript; Available in PMC 2026 Apr 7. (PMC7618982; doi:10.1016/j.jhin.2025.02.002)

**Supplementary material**

**Sinkbug consortium author list and affiliations**

| Name | Surname | Hospital |
| --- | --- | --- |
| Shrikant | Ambalkar | King’s Mill Hospital, Sutton-in-Ashfield, UK |
| Kashif | Aziz | Leicester Royal Infirmary, Leicester, UK |
| Vhairi | Bateman | Aberdeen Royal Infirmary, Aberdeen, UK |
| Kevin | Bertram | Cumberland Infirmary, Carlisle, UK and West Cumberland Hospital, Whitehaven, UK |
| Emily | Broadwell | Leeds Children Hospital, Leeds, UK |
| Dominique | Chaput | Glasgow Royal Infirmary, Glasgow, UK; Queen Elizabeth University Hospital, , Glasgow, UK; Royal Alexandra Hospital, Paisley, UK |
| Rinkuvijay | Chourasia | University Hospital Coventry, Coventry, UK |
| Carly | Clare | Queens Medical Centre, Npttingham, UK |
| Alan | Cordey | University Hospital Llandough, Llandough, UK |
| Chris | Darlow | Manchester Royal Infirmary, Manchester, UK; Royal Liverpool University Hospital, Liverpool, UK |
| Michael | Dibbens | St Thomas' Hospital, London, UK |
| Vilde | Dietz | Nuffield Department of Medicine, University of Oxford, Oxford, UK |
| Catherine | Edwards | Balfour Hospital, Kirkwall, UK |
| Vicki | Fleming | Queens Medical Centre, Nottingham, UK |
| Simon | Goldenberg | St Thomas' Hospital, London, UK |
| Callum | Goolden | Royal Preston Hopsital, Preston, UK |
| Clive | Graham | Cumberland Infirmary, Carlisle, UK and West Cumberland Hospital, Whitehaven, UK |
| Anna | Green | Northern General Hospital, Sheffield, UK |
| Hudson | Guyver | James Paget Hospital, Great Yarnouth, UK |
| Fenella | Halstead | Hereford County Hospital, Hereford, UK |
| Tom | Harrison | Northern General Hospital, Sheffield, UK |
| Lauren | Hookham | John Radcliffe Hospital, Oxford, UK; Stoke Mandeville Hospital, Aylesbury, UK |
| Katie | Hopkins | UKHSA Colindale, London, UK |
| Susan | Hopkins | UKHSA Colindale, London, UK |
| Gareth | Hughes | Birmingham Heartlands Hospital, Birmingham, UK |
| Wendy | Ibbotson | Northern General Hospital, Sheffield, UK |
| Rhys | John Davies | University Hospital Wales, Cardiff, UK; University Hospital Llandough, Llandough, UK |
| Alison | Johnson | Hereford County Hospital, Hereford, UK |
| Claire | Johnston | Morriston hospital, Swansea, UK |
| Eben | Jones | Southmead Hospital, Bristol, UK |
| Sharon | Jose | Ninewells Hospital, Dundee, UK |
| Justin | Joy | James Paget Hospital, Great Yarnouth, UK |
| Aleksandra | Marek | Glasgow Royal Infirmary, Glasgow, UK; Queen Elizabeth University Hospital, , Glasgow, UK; Royal Alexandra Hospital, Paisley, UK |
| William | Matlock | Nuffield Department of Medicine, University of Oxford, Oxford, UK |
| Damian | Mawer | York Hospital, York, UK |
| Alex | May | University Hospital Llandough, Llandough, UK |
| Ciaran | Mooney | Royal Victoria Hospital, Belfast, UK |
| Alison | Muir | Royal Preston Hopsital, Preston, UK |
| Clemency | Nye | University Hospital Wales, Cardiff, UK; University Hospital Llandough, Llandough, UK |
| Ijeoma | Okoliegbe | Aberdeen Royal Infirmary, Aberdeen, UK |
| Benjamin | Parcell | Ninewells Hospital, Dundee, UK |
| Suzanna | Paterson | Royal Victoria Hospital, Belfast, UK |
| Emma | Pritchard | Nuffield Department of Medicine, University of Oxford, Oxford, UK |
| Phuong | Quan | Nuffield Department of Medicine, University of Oxford, Oxford, UK |
| Stuart | Reid | Ninewells Hospital, Dundee, UK |
| Nurfarah | Sabtu | University Hospital Coventry, Coventry, UK |
| Avinandan | Saha | King’s Mill Hospital, Sutton-in-Ashfield, UK |
| Kavita | Sethi | Leeds Children Hospital, Leeds, UK |
| Shanya | Sivakumaran | Morriston hospital, Swansea, UK |
| Asanka | Tennegedara | King’s Mill Hospital, Sutton-in-Ashfield, UK |
| Sarah | Walker | Balfour Hospital, Kirkwall, UK |
| Mariela | Webs | Nuffield Department of Medicine, University of Oxford, Oxford, UK |
| Becky | Wilson | Balfour Hospital, Kirkwall, UK |
| Lorna | Wilson | Balfour Hospital, Kirkwall, UK |
| Gemma | Winzor | Birmingham Heartlands Hospital, Birmingham, UK |
| Nick | Wong | Stoke Mandeville Hospital, Aylesbury, UK |
| Dominic | Worku | Morriston hospital, Swansea, UK |
|  |  |  |

**Table S1. Guidance/published evidence supporting sink specific design features to mitigate pathogen transmission risk.**

| **Sink design feature** | **Reference (from main text)** | **Recommendations from cited study/guidance** | **Characteristics identified in this study** |
| --- | --- | --- | --- |
| Distance of sink to patients | 9, 20, 24 | Suggest >1m away from patients as this represents the approximate extent of the measured splatter zone | Distance in metres recorded |
| Sink basin material type | 9 | Plastic encourages biofilm formation | Ceramic  Plastic  Stainless steel |
| Sink basin depth | 9, 18 | Shallow sink basins associated with greater risk of splatter | Not assessed |
| Presence of plug | 18 | Plugs should not be allowed in non-bathroom basins | Yes  No |
| Presence of overflow | 18 | Overflows are not recommended as they constitute an infection control risk | Yes  No |
| Presence of strainer | 24, 27 | Recommendation is not to have a strainer | Yes  No |
| Location of drain with respect to tap outlet | 9, 15, 18, 20, 21 | Tap outlet should be positioned offset from drain so that the water stream does not discharge onto the basin’s waste outlet | Under the taps  Offset from taps |
| Drainhole position | 21 | Horizontal drainhole positioning associated with less splatter | Horizontal  Vertical |
| Presence of a basin fin | 22 | A ceramic fin in the basin reduces splatter | Yes  No |
| Location of taps | 18 | Sinks used in clinical areas should have wall-mounted tap holes | Wall-mounted  Not wall-mounted |
| Method of tap operation | 18 | Lever-action or sensor-associated taps are ideal | Long-lever  Short-lever  Other |
| Shape of tap outlet spout | 18 | Should not be a goose-neck design as these do not typically empty after use | Gooseneck  Markwik 21+  Other |
| Tap point-of-use filters | 9 | Evidence around these clearer for mitigating against pathogens in the incoming water supply; they may leak, get contaminated, or alter flow rates affecting microbial growth | Yes  No |
| Faucets with aerators or flow modulators | 9, 18 | Recommended not to have these | Not assessed |
| Items surrounding basin | 9, 27 | Items within the splatter field can become contaminated with splatter from colonised strainers/basins | Yes  No |
| Sink misuse | 9, 11 | Nutrients, antibiotics, patient waste etc. should not be disposed of down handwashing/patient-facing sinks | Not assessed |
| Sink cleaning strategy | 9 | Optimal cleaning strategies remain unclear | Not assessed |
| Water splatter when taps turned on | 9, 27 | Items within the splatter field can become contaminated with splatter from colonised strainers/basins | Yes  No |
| Water flow and pooling/drainage | 9, 21 | Inadequate drainage and water pooling contributes to greater splatter risk | Not assessed |

**Supplementary Figure S1. Results of sink trap biochemistry evaluations, stratified by ward type,** including ICUs (n=111 sinks), medical wards (n=92 sinks) and surgical wards (n=84 sinks).


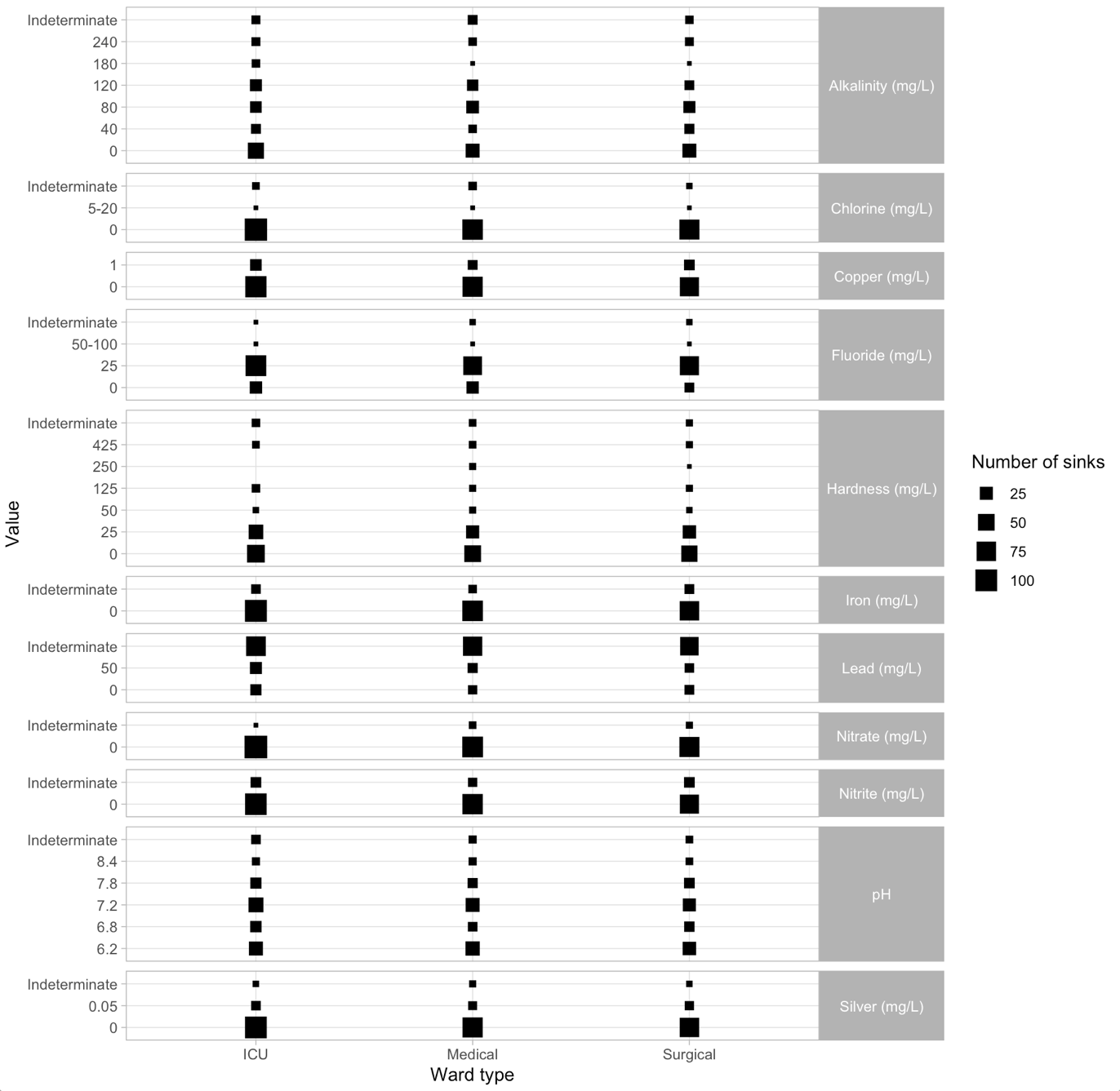


**Supplementary Figure S2. Results of sink trap biochemistry evaluations, stratified by sink location,** including medicines/drug preparation rooms (n=77 sinks), patient bays (n=97 sinks), patient side-rooms (n=25 sinks) and sluice rooms (n=88 sinks).


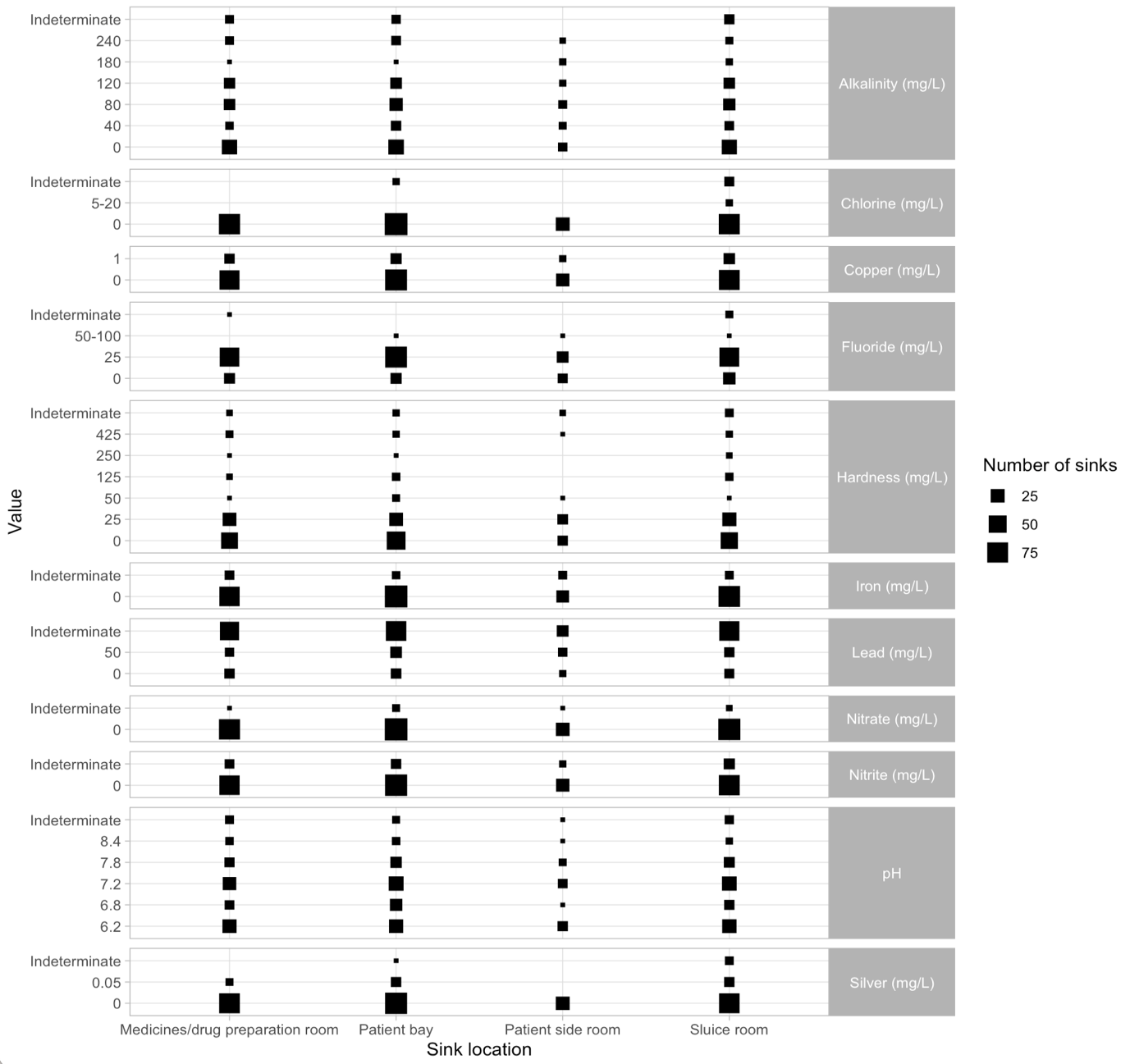

Supplement: Supplementary material [file EMS213008-supplement-Supplementary_material.docx]
